# Analysis of Field of View for a Moon-Based Earth Observation Multispectral Camera

**DOI:** 10.3390/s24216962

**Published:** 2024-10-30

**Authors:** Zhitong Yu, Hanlin Ye, Mengxiong Zhou, Feifei Li, Yin Jin, Chunlai Li, Guang Liu, Huadong Guo

**Affiliations:** 1 Qian Xuesen Laboratory of Space Technology, China Academy of Space Technology, Beijing 100094, China; yztgeo@126.com; 2 International Center for Climate and Environment Science, Institute of Atmospheric Physics, Chinese Academy of Sciences, Beijing 100029, China; 3 Key Laboratory of Digital Earth Science, Aerospace Information Research Institute, Chinese Academy of Sciences, Beijing 100094, China; zhoumengxiong23@mails.ucas.ac.cn (M.Z.); jinyin22@mails.ucas.ac.cn (Y.J.); liuguang@radi.ac.cn (G.L.); hdguo@radi.ac.cn (H.G.); 4 Key Laboratory of Space Active Opto-Electronics Technology, Shanghai Institute of Technical Physics, Chinese Academy of Sciences, Shanghai 200083, China; lifeifei@mail.sitp.ac.cn (F.L.); lichunlai@mail.sitp.ac.cn (C.L.)

**Keywords:** Moon-based Earth observation multispectral camera, field of view, Earth’s apparent diameter, pointing accuracy, pointing adjustment temporal interval, solar intrusion

## Abstract

A Moon-based Earth observation multispectral camera provides a unique perspective for observing large-scale Earth phenomena. This study focuses on the analysis of the field of view (FOV) for such a sensor. Unlike space-borne sensors, the analysis of the FOV for a Moon-based sensor takes into account not only Earth’s maximum apparent diameter as seen from the lunar surface but also the Earth’s and the solar trajectory in the lunar sky, as well as the pointing accuracy and pointing adjustment temporal intervals of the turntable. Three critical issues are analyzed: (1) The relationship between the Earth’s apparent diameter and the Earth’s phase angle is revealed. It is found that the Earth’s maximum apparent diameter encompasses the Earth’s full phase, suggesting the FOV should exceed this maximum. (2) Regardless of the location on the lunar surface, a sensor will suffer from solar intrusion every orbital period. Although the Earth’s trajectory forms an envelope during an 18.6-year cycle, the FOV should not be excessively large. (3) To design a reasonable FOV, it is necessary to consider both the pointing accuracy and pointing adjustment temporal interval comprehensively. All these insights will guide future Moon-based Earth observation multispectral camera design.

## 1. Introduction

The Moon, as the only natural satellite of the Earth [1], can serve as an Earth observation platform. The history of Moon-based Earth observations can be dated to 1972, when the astronauts of Apollo 16 used a far ultraviolet spectrograph to capture 178 images of Earth, and it was the first time seeing the Earth from the Moon [2]. With the development of space science and technology, more and more countries and international organizations gained great interest in conducting Moon-based Earth observations [3,4,5,6].

As the first lunar probe of the European Space Agency (ESA) and the first research project in the ESA’s series of Small Missions Advanced Technology Research (SMART), SMART-1 was launched in 2003 to validate new propulsion systems, followed by lunar exploration. It carried Advanced Moon micro-Imager Equipment (AMIE), which took spectacular pictures of the Earth during a lunar eclipse for the first time [7]. SELenological and ENgineering Explorer KAGUYA (SELENE), developed in cooperation with the Japan Aerospace Exploration Agency (JAXA) and Nippon Hoso Kyokai (NHK), was launched in 2007 with the scientific objectives of obtaining data on the Moon’s and Earth’s origin and evolution and developing the technology for future lunar exploration. On 10 February 2009, KAGUYA successfully captured a video of the Earth rising on the lunar horizon with its onboard high-definition television (HDTV) camera [8]. The Extreme Ultraviolet Camera (EUVC) mounted on the rover of Chang’E-3 is the first dedicated sensor for Earth observation on the lunar surface by China [9].

In 2006, the National Aeronautics and Space Administration (NASA) held a lunar Earth observatory workshop. Hamill [10] proposed a robotic Earth observatory on the lunar surface, equipped with a telescope, a diffraction grating with an associated Charge-Coupled Device (CCD) array, a CCD camera, and a radiometer, enabling the collection of scientifically significant atmospheric data over extended periods. Johnson et al. [11] suggested a visible/near-infrared hyperspectral instrument viewing the Earth from a Moon-based platform, aimed at observing atmospheric composition and Earth’s bidirectional reflectivity distribution function for climate research and other scientific objectives. In 2016, the International Geoscience and Remote Sensing Symposium (IGARSS) organized a special session on Moon-based Earth observations, where scientists from China, the United States, and Europe discussed pioneer research on Moon-based Earth observations in terms of scientific objectives and observation methods [12,13,14]. In the subsequent years, scholars from around the world have conducted discussions on the scientific objectives, observation geometry, and imaging mechanisms for Moon-based Earth observations [15,16,17,18,19].

The Earth observation geometry for a Moon-based platform is significantly different from both geostationary orbit (GEO) satellites and the platform at the Sun-Earth Lagrange L1 point. A Moon-based platform can observe almost the entire Earth hemisphere, whereas GEO platforms do not have a wide enough FOV to observe the polar regions. Additionally, sensors equipped on GEO satellites typically use scanning methods to mosaic together a disk [20]. Compared to the case at the Earth–Sun L1 point, a sensor equipped on the lunar surface can view the Earth’s disk under different phase angles [5,21].

The multispectral camera is a prevalent sensor for Earth observation, with the potential for a Moon-based version to capture comprehensive full-disk spectral imagery. While existing literature primarily addresses geometric imaging models [22] and temporal sampling intervals [23], discussions on the FOV for such multispectral cameras on the lunar surface are insufficient. A detailed analysis of the FOV for a Moon-based Earth observation multispectral camera is essential. Low Earth orbit (LEO) satellite platforms, which typically operate at lower altitudes, often employ linear array push-broom or whisk-broom scanning methods to acquire strip multispectral image data [24,25]. The design of the swath width requires a trade-off with spatial resolution. The current pursuit is the design of wide-swath, high-resolution multispectral cameras. The FOV of multispectral cameras on GEO satellites is approximately 1°, and they acquire Earth data through scanning methods [26,27]. The limiting factor for these sensors is mainly the complexity of the optical system. The Earth Polychromatic Imaging Camera (EPIC), located at the Sun–Earth Lagrange L1 point aboard the Deep Space Climate Observatory (DSCOVR), has an FOV of approximately 0.6°, slightly larger than the Earth’s apparent diameter at this location [21]. However, it can observe the entire sunlit side of the Earth without the need to consider the effects of stray light. Additionally, it only needs to adjust its attitude to consistently point towards the Earth, without concerns of obstructions.

Deploying sensors on the Moon presents unique challenges compared to all the aforementioned platforms. Existing sensors on the lunar surface are primarily focused on observing Earth’s outer atmosphere, with a wide FOV that does not provide an effective reference for Earth observation sensors [28,29]. The design of the FOV should take into account not only the Earth’s apparent diameter as viewed from the lunar surface but also the complexity of the sensor’s design and the spatial resolution of the anticipated data acquisition. Atmospheric refraction induces the bending of light, potentially resulting in slight deviations in the Earth’s apparent diameter. A well-conceived FOV design can simplify the complexity of optical and thermal control systems, optimize parameter design, and enhance the capability for Earth observation. Furthermore, the selection of FOV must consider the Earth–Sun–Moon geometric relationships and the turntable’s pointing control capabilities to accurately target the Earth. During the design process, it is essential to consider the variations in the Earth’s and the solar trajectory in the lunar sky from different locations on the Moon, as well as the pointing accuracy of the turntable.

Distinguished from prior research, this study focuses on merging theoretical insights with practical engineering applications. By identifying the factors that influence FOV design and summarizing the patterns of Earth and solar trajectory changes as observed from different locations on the lunar surface, this study offers valuable insights for engineers. There are three main contributions. First, the Earth’s apparent diameter, accounting for atmospheric refraction, is deduced. We also examined the relationship between the Earth’s apparent diameter and the corresponding phase to explain why, even with the presence of the phase, the FOV must still account for the Earth’s maximum apparent diameter. Second, the characteristics of the Earth’s and the solar trajectory in the lunar sky are analyzed. This explains the varying effects of solar intrusion at different locations on the lunar surface, and it reveals the magnitude and direction of the Earth’s movement within the FOV. These factors will all serve as constraints in the design of the FOV. Third, the impacts of pointing error on the determination of the FOV are investigated, and the analysis also examines how pointing errors and pointing adjustment temporal intervals affect the FOV.

## 2. Scientific Goals for a Moon-Based Earth Observation Multispectral Camera

Moon-based Earth observations offer several characteristics: (1) Long-term observations. Unlike artificial satellites, which typically have a lifespan of a few years to decades, the Moon has a history of 4.5 billion years and will continue to exist in the future, making it a ‘permanent’ platform [30]. Long-term observations can reduce calibration errors among sensors, forming reliable time series data to support global change studies. (2) Hemisphere-scale observations. A Moon-based sensor has the capability to cover the whole Earth’s disk at various wavelengths. The vast space of the Moon can equip a variety of sensors, achieving a comprehensive acquisition of Earth’s spheres and forming a three-dimensional observation [31]. (3) Unique observations. The Moon is the primary source of Earth’s tidal forces, which significantly impact atmospheric, oceanic, and solid Earth tides. Observing from the Moon allows for a more direct study of how these tidal forces affect Earth’s tidal dynamics, including the periodic fluctuations in tides and their relationship with the relative positions of the Moon and the Sun. In addition, the Moon’s stability facilitates the deployment of multiple sensors, such as synthetic aperture radars (SARs), creating stable baselines that enhance interferometric precision and aid in measuring solid tides [5].

The main scientific goals of a Moon-based Earth observation multispectral camera are the following:

(1) To estimate the Earth’s outgoing radiation at the top of atmosphere (TOA): The full-disk view and multi-angle capabilities of a Moon-based Earth observation multispectral camera can provide hemispherical-scale, multi-angle, and multispectral observational data for Earth’s outgoing radiation observations [5].
(2) To carry out comprehensive observations on the large-scale phenomena for studying climate change: Most of the Earth’s phenomena have the characteristics of large-scale and rapid changes. A Moon-based Earth observation multispectral camera can achieve comprehensive observations at various wavelengths that maintain both spatial consistency and temporal continuity [32].
(3) To observe the Earth’s atmosphere and surface characteristics for informing exoplanet studies: The Earth’s atmospheric and surface characteristics render it an ideal reference for the study of signatures on extrasolar planets. A Moon-based Earth observation multispectral camera can offer an integrated characterization of reflected light, and thermal self-emission, to characterize signatures of the Earth, which can aid in providing samples for the search for habitable exoplanets [33,34,35,36,37,38]. For example, the observation of self-emission can be utilized to characterize terrestrial planets. This is due to the detectability of diagnostic atmospheric species at temperatures that support habitability, along with their capacity to refine theoretical predictions of equilibrium temperatures. Many scholars have proposed, innovatively, using similar platform data, to study Earth as if it were an exoplanet [39,40,41,42].

## 3. Materials and Methods

**Figure 1:**
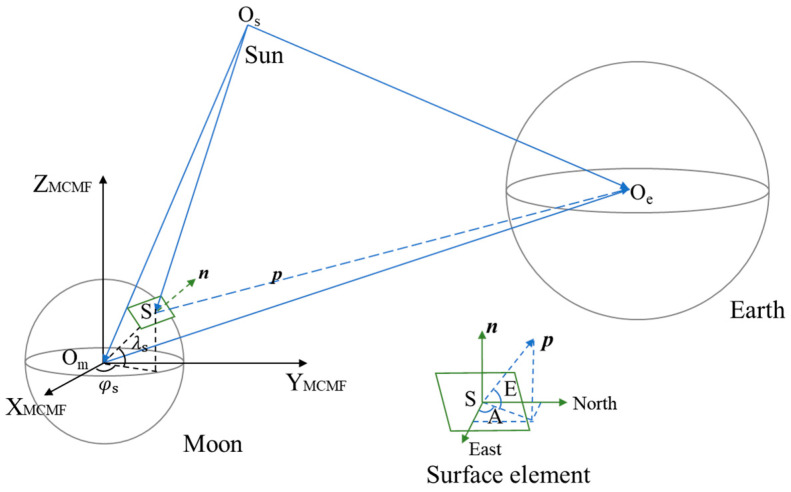
Illustration of the Moon-based Earth observation.

The analysis of FOV for a Moon-based Earth observation multispectral camera requires the geometric relationship between the Earth, the Moon, and the Sun. Figure 1 shows the observation geometry. The symbols O_m_, O_s_, and O_e_ denote the barycenter of the Moon, the Earth and the Sun, respectively. These data must be obtained from the ephemeris. The sensor position S is determined by the lunar latitude and longitude. The vector *n* represents the normal vector at the location where the sensor is deployed. The Earth’s pointing vector *p* is characterized by the elevation angle *E* and the azimuth angle *A*. In this section, the used data including the planetary ephemeris and Earth orientation parameters (EOPs) are first introduced. Subsequently, the calculation method for the Earth’s and the solar position in the lunar sky is proposed to reveal the characteristics of their trajectories, and then the relationship between the Earth and the Sun in the lunar sky can be provided. Finally, the calculation method for Earth’s apparent diameter considering the atmospheric refraction is provided.

### 3.1. Data

#### 3.1.1. Planetary Ephemeris

Planetary ephemeris is an important data source in studying the geometric relationships between the Moon, the Earth, and the Sun in Moon-based Earth observations. Numerical integration methods are employed and continuously update parameters with the latest observational data, ensuring the output of required data with extremely high precision over a span of several centuries. The mainstream planetary ephemerides currently in use are those from the Jet Propulsion Laboratory (JPL) in the United States [43], the Paris Observatory in France [44], and the Institute of Applied Astronomy of the Russian Academy of Sciences [45]. This study selected Development Ephemeris (DE), with the chosen version being DE430 [43]. It provides Chebyshev polynomial coefficients, which, upon calculation, yield positional information at different times. In addition, the lunar libration is also given in the ephemeris. The position of the Moon is generally defined in the geocentric celestial reference system (GCRS), while the positions of the Sun and Earth are generally defined in the International Celestial Reference System (ICRS). Therefore, to describe the geometric relationship between the Sun, Earth, and Moon, it is necessary to unify these three celestial bodies within the same coordinate system [22].

#### 3.1.2. EOPs

EOPs are a set of parameters that describe the orientation of the Earth’s rotation axis in space and the variations in the Earth’s rotation speed. These parameters are crucial for accurately determining the Earth’s attitude within a Moon-based FOV. The EOP mainly includes the following types of parameters: polar motion, Earth rotation rate (UT1-UTC), precession, and nutation. The measurement and prediction of EOP parameters are two of the main tasks of the International Earth Rotation and Reference Systems Service (IERS) [46]. The Sub-bureau for Rapid Service and Predictions of Earth Orientation Parameters of the IERS is situated at the United States Naval Observatory in Washington, D.C., USA. The IERS collects data and calculates EOP parameters through a global observation network, such as Very Long Baseline Interferometry (VLBI), Global Positioning System (GPS), Galileo, Global Navigation Satellite System (GNSS), and Satellite Laser Ranging (SLR). In coordinate system transformations, EOPs primarily assist in the conversion between the ICRS and the International Terrestrial Reference System (ITRS).

### 3.2. Earth and Solar Position Calculation in the Lunar Sky

**Figure 2:**
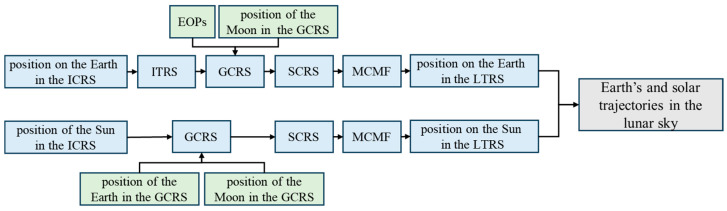
Calculation process of the Earth’s and solar trajectories in the lunar sky.

To investigate the Earth’s and the solar trajectory as observed from different locations on the lunar surface, it is necessary to employ coordinate transformations to calculate the coordinates of the Earth and the Sun in the Lunar Topocentric Reference System (LTRS). To illustrate the calculation process more clearly, Figure 2 presents the sequence of coordinate system transformation and the calculation of the Earth’s and the solar trajectories in the lunar sky.

The position on Earth in the LTRS can be written as follows:

(1) $$p_{LTRS}=R_{MCMF}^{LTRS}R_{SCRS}^{MCMF}T_{GCRS}^{SCRS}R_{ITRS}^{GCRS}p_{ITRS}$$

where $p_{ITRS}$ represents the position on Earth in the ITRS. The matrix $R_{ITRS}^{GCRS}$, based on EOPs, facilitates the coordinate transformation from ITRS to the GCRS. The matrix $T_{GCRS}^{SCRS}$ achieves the coordinate transformation from GCRS to the Selenocentric Celestial Reference System (SCRS) by translating the origin from the Earth’s barycenter to the lunar barycenter. The matrix $R_{SCRS}^{MCMF}$, based on lunar libration provided by the planetary ephemeris, realizes the coordinate transformation from SCRS to the Moon-fixed Coordinate System (MCMF). Lastly, the matrix $R_{MCMF}^{LTRS}$ accomplishes the coordinate transformation from MCMF to the LTRS based on the geographical latitude and longitude of the observation point on the lunar surface. Detailed transformations can be found in [22,23].

Similarly, the position of the Sun in the LTRS can be written as follows:

(2) $$s_{LTRS}=R_{MCMF}^{LTRS}R_{SCRS}^{MCMF}T_{GCRS}^{SCRS}s_{GCRS}$$

where $s_{GCRS}$ represents the position coordinates of the Sun in GCRS, and can be directly derived from the planetary ephemeris.

In order to reasonably represent the Earth’s and the solar position in the lunar sky, this study utilizes the azimuth and elevation angles as two parameters, thereby ensuring consistency in expression with the sensor’s line-of-sight vector. Under this definition, the azimuth angle *γ* is set with the east direction as 0°, with counterclockwise rotation being positive; the elevation angle is set with the horizontal direction as 0°, with upward direction being positive.

More ingeniously, according to the definition that the positive direction of the MCMF x-axis is the mean direction towards the Earth, the mean elevation and azimuth angles can be further expressed as functions of the lunar latitude $\lambda_{s}$ and longitude $\varphi_{s}$:

(3) $$A=\tan^{-1}\left(\frac{\sin\lambda_{s}}{\tan\varphi_{s}}\right)$$

(4) $$E=\sin^{-1}\left(\cos\lambda_{s}\cos\varphi_{s}\right)$$

This facilitates the revelation of the distribution characteristics of the elevation and azimuth angles at different locations on the lunar surface.

### 3.3. Earth’s Apparent Diameter Calculation

The Earth’s apparent diameter, as observed from the Moon, refers to the size of the Earth’s diameter as it appears from the lunar surface. It is primarily influenced by the Earth–Moon distance, and in addition is also affected by the refraction of the Earth’s atmosphere. The formula to calculate the Earth’s apparent diameter is as follows:

(5) $$D_{apparent}={2\sin}^{-1}\frac{R_{e}+h}{d}$$

where *R*_e_ is the Earth’s radius; the parameter *h* is the height of the TOA, approximately 100 km; and the parameter *d* is the Earth–Moon distance.

Further, for precisely calculating the Earth’s apparent diameter observed from the Moon, one must also consider the effects of the Earth’s atmospheric refraction. According to the conclusions of Hohenkerk et al. [47], the atmospheric refractive index *n* can be considered as a single-variable function of height. By applying Snell’s law to a spherically symmetric atmosphere, the angle *ξ* by which the line-of-sight vector is deflected due to atmospheric refraction at any given location can be expressed by the following equation:

(6) $$\xi =-\int_{0}^{z_{0}}\frac{r\;dn/dr}{n+r\;dn/dr}dz$$

where *r* represents the distance between any point on the propagation path of the line-of-sight vector and the Earth’s barycenter, *z* denotes the zenith angle of specific position on Earth, and *z*_0_ corresponds to the maximum zenith angle of the Earth. By setting *z*_0_ = 90°, the deflection angle *ξ* corresponding to the line-of-sight vector tangent to the point on Earth at any position along the propagation path can be calculated.

Subsequently, the deflection distance *h*_1_ can be obtained as follows:

(7) $$h_{1}=\int_{0}^{z_{0}}\frac{nr}{n+r\;dn/dr}\frac{\xi }{\sin z}dz$$

Therefore, an expression for the increment in the Earth’s apparent diameter due to atmospheric refraction can be expressed as follows:

(8) $$\alpha =\frac{\sin^{-1}\left[\left(r_{e}+h+h_{1}\right)/d\right]}{\sin^{-1}[\left(r_{e}+h\right)/d]}-1$$

## 4. Results

To design of the FOV for a Moon-based Earth observation multispectral camera, an analysis of the Earth’s apparent diameter over an 18.6-year period, which corresponds to the lunar nodal cycle, is essential. To ensure full-disk observation of the Earth, the FOV must exceed the Earth’s maximum apparent diameter during the lunar nodal cycle, which is the fundamental basis for determining the size of the FOV. Additionally, the geometric relationships between the Sun, Earth, and Moon are crucial, as the complex Sun–Earth–Moon geometry can lead to solar intrusion during observations, affecting the quality of the data. Studying the trajectories of the Earth and the Sun in the lunar sky can reveal the regularity of solar intrusion, thus laying the foundation for the design of the FOV. Finally, further considerations are given to analyze the impact of pointing errors and adjustment temporal intervals on the determination of the FOV.

### 4.1. Analysis of the Earth’s Apparent Diameter

Earth’s apparent diameter is the base of the design of the FOV for a Moon-based multispectral camera. As detailed in Section 2, the Earth’s apparent diameter is influenced by the distance from the sensor to the Earth, as well as the impact of atmospheric refraction. Due to complex Sun–Earth–Moon geometric relationship, the Earth–Moon distance varies periodically over the course of the 18.6-year lunar nodal cycle.

**Figure 3:**
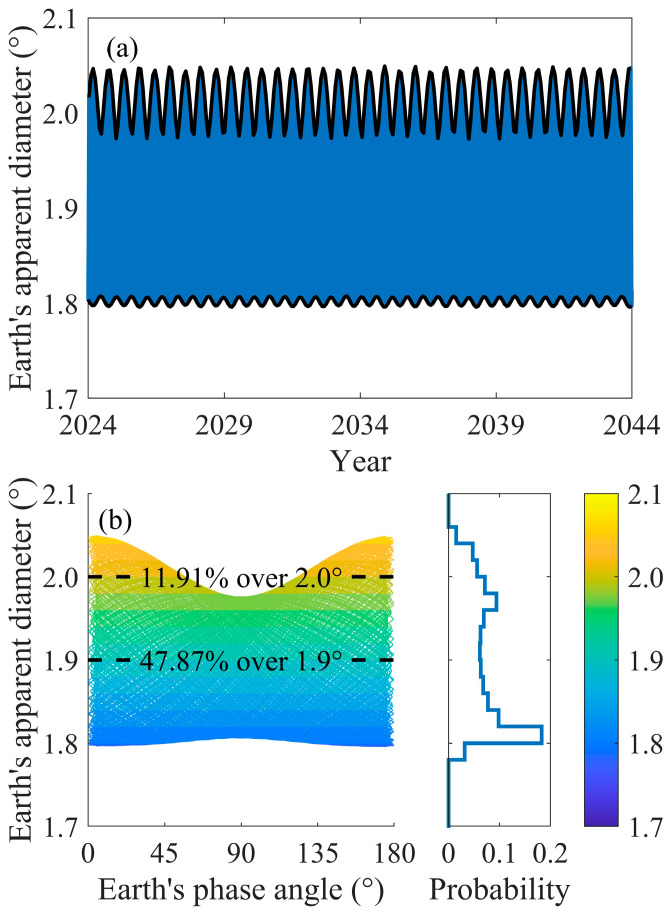
(**a**) Earth’s apparent diameter variations from 2024 to 2044. (**b**) Earth’s apparent diameter with variations in Earth’s current phase angle from 2024 to 2044.

Figure 3 shows the variation in the Earth’s apparent radius from 2024 to 2044. Despite the varying observational performance from different locations on the lunar surface, such as visibility capabilities to the Earth or lunar topographical influences, the differences in Earth’s apparent diameter observed from various points on the lunar surface are minimal, as the lunar radius is only one-fourth of a percent of the Earth–Moon distance. The variation cycle of the Earth–Moon distance is approximately 27.3 days, which is also known as a sidereal month. During this period, the Moon moves from its perigee to its apogee and back to its perigee, with its speed being faster at the perigee than at the apogee. Under these conditions, the Earth’s apparent diameter also has a corresponding period, varying from approximately 1.80° to 2.05°. Additionally, there is an approximately semi-annual period for the perigee and apogee, which also leads to the periodic variation in the Earth’s apparent diameter.

We also investigated the correlation between the Earth’s phase and its apparent diameter. Similarly to observing the Moon from the Earth, the waxing and waning of the Earth, which is termed the Earth’s phase, can also be observed from the Moon. The full Earth denotes to the Earth’s phase angle of 180°, while the ‘new Earth’ corresponds to an angle of 0°. The Earth’s apparent diameter exhibits a symmetrical distribution with respect to the Earth’s phase angle at 90°. The motivation for this analysis is that a smaller FOV would alleviate the complexity of the multispectral camera’s optical system design; thus, minimizing the FOV to the greatest extent possible is a goal in such sensor design. Given the existence of different Earth phases and the fact that the sensor primarily focuses on the visible light spectrum, a plausible idea emerges: when the Earth’s phase is not full, adjustments to the pointing could be made to keep the Earth’s entire sunlit portion within the FOV. To verify the feasibility of this idea, it is necessary to reveal the relationship between the Earth’s phase angle and Earth’s apparent diameter. We have counted the probability distribution of Earth’s phase angle as it varies with Earth’s apparent diameter. As can be seen from Figure 3b, comparatively, the Earth’s apparent diameter falls within the range of 1.80° to 1.82° for a significant portion of the time, during which the ‘new Earth’ and ‘full Earth’ phases exhibit a smaller range of phase angles than the other Earth phases. Over 50% of the time, Earth’s apparent diameter is below 1.90°. It can be observed that although the time when Earth’s apparent diameter exceeds 2.00° accounts for approximately 11.91%, in most of those cases, it corresponds to the ‘new Earth’ and ‘full Earth’ phases. Consequently, the design of the FOV must still take into account the observation of the Earth’s full disk.

**Figure 4:**
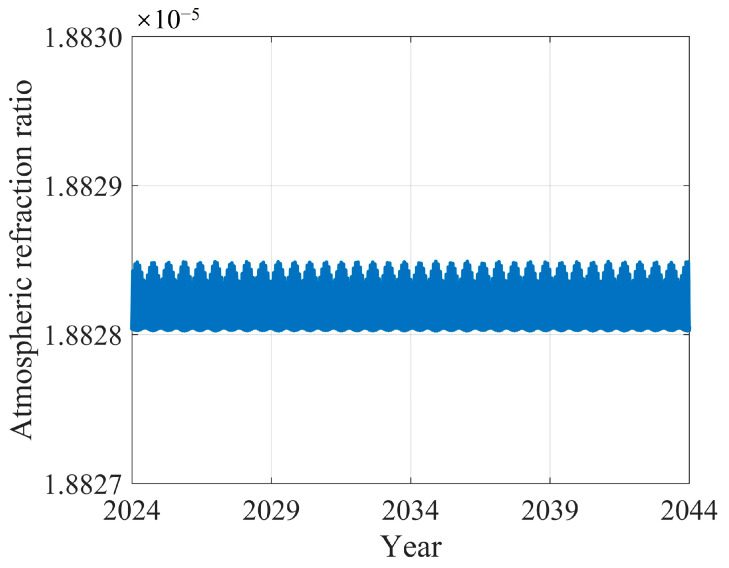
Atmospheric refraction ratio variations from 2024 to 2044.

An additional factor to consider is the effect of atmospheric refraction on the Earth’s apparent diameter. The Earth’s atmosphere can cause light rays to refract during their propagation. Since a Moon-based sensor can observe an entire hemisphere of the Earth, there is a possibility that the Earth’s apparent diameter may be slightly magnified. In the case of LEO and GEO satellites, atmospheric refraction can cause geolocation errors in the image, particularly at the edges, which has a significant impact on high-resolution remote sensing. In the Moon-based case, where a hemisphere of the Earth is captured, the effect of atmospheric refraction at the edges is amplified due to the oblique path of light through the atmosphere. According to Garfinkel [48], the light ray emitted from the Earth’s surface at a zenith angle of 90° experiences a deflection of 0.57° during its propagation. This deflection effect causes the observation point on the Earth’s disk edge to move approximately 96 m perpendicular to the line-of-sight vector. This leads to an increase in the Earth’s apparent diameter by about 2.9 × 10^−5^°. To analyze how the atmospheric refraction effect varies over time on the Earth’s apparent diameter observed from a Moon-based platform, the proportion by which the Earth’s apparent diameter increases due to atmospheric refraction is defined as the atmospheric refraction ratio. As can be seen from Figure 4, the range of the atmospheric refraction ratio is 1.88 × 10^−5^°, which is inversely proportional to the Earth–Moon distance. The ratio refers to the projected movement of Earth’s edge observation points, perpendicular to the line-of-sight vector considering atmospheric refraction, relative to the Earth’s radius. As the Earth–Moon distance increases, the line-of-sight vector from the Moon-based sensor to the points at the Earth’s disk edge become more parallel to each other, causing the edge points to move a greater distance in the direction perpendicular to the line-of-sight vector, thereby leading to an increase in the ratio. Consequently, in the Moon-based case, the impact of atmospheric refraction on the Earth’s apparent diameter is extremely weak and can be neglected.

### 4.2. Earth and Solar Trajectory Analysis Across the Lunar Sky

Unlike the LEO satellite, whose pointing towards the Earth’s barycenter is maintained by changing the satellite’s attitude, a Moon-based Earth observation sensor uses a two-dimensional turntable, similar to ground-based telescopes, to point at the Earth’s barycenter. Due to limitations in energy and rotation mechanism wear, the two-dimensional turntable cannot achieve continuous real-time tracking; therefore, it is necessary to establish a time interval for tracking steps, and within this interval, it must be ensured that the Earth’s whole disk remains within the FOV. The complex relative positions of the Sun, Earth, and Moon necessitate an understanding of their trajectories within the lunar sky, which is essential for characterizing the FOV of the Moon-based Earth observation multispectral camera. A question arises: does the Earth’s trajectory across the lunar sky follow the same east-to-west pattern as the solar trajectory across the Earth’s sky, or is it concentrated in a particular direction in the lunar sky? Furthermore, is it possible to design an FOV large enough to encompass the Earth’s entire trajectory across the lunar sky throughout the entire 18.6-year cycle?

**Figure 5:**
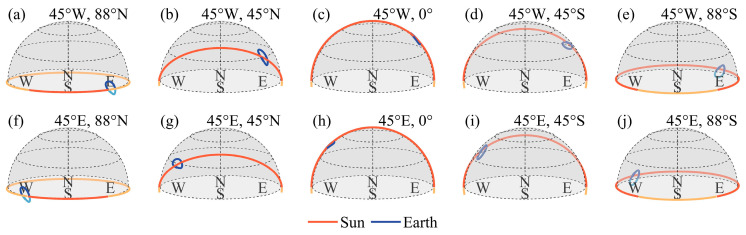
The Earth’s and the solar trajectory in the lunar sky at different positions on the lunar surface.

The Moon is a celestial body, and the Earth varies in the lunar sky, being observed from different locations on the lunar surface. Figure 5 show the Earth’s and the solar trajectory across the lunar sky at different locations on the lunar surface. Previous studies indicated that the observation time window from different locations on the Moon can generally be categorized into regions where the Earth is perpetually visible and those where the Earth is not always visible [49]. Among them, the regions where Earth is not always visible are primarily located at the limb of the lunar disk or in the polar regions. We selected 88° S and 88° N to simulate the Earth’s and the solar trajectory in the lunar sky. Due to the large latitudinal span of regions where Earth is perpetually visible, this study selected the equator and 45° N and 45° S as representative examples. By comparing the Earth’s trajectory as viewed from regions of perpetual visibility and partial visibility on the lunar surface, it is found that the Earth’s motion in the lunar sky has been more clearly illustrated. The placement of sensors at different lunar latitudes determines the range of variation in the elevation angle of Earth’s trajectory, with higher elevation angles approaching the equator. The placement of sensors on the lunar longitude is associated with the range of azimuth angles for observing Earth. Sensors located in the eastern hemisphere of the Moon will observe Earth appearing in the western part of the lunar sky.

**Figure 6:**
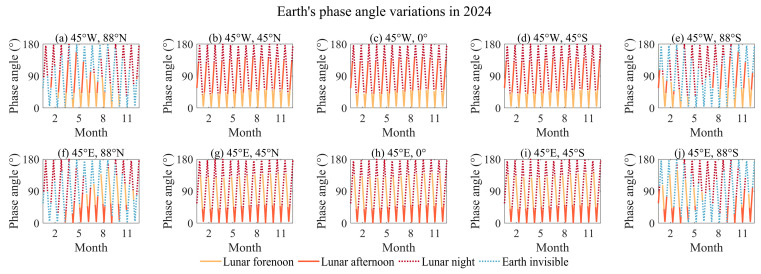
The Earth’s phase angle variations at different positions on the lunar surface over one year.

Compared to LEO and GEO satellites, due to the complex Earth–Moon–Sun relationships, observations of the Earth from the lunar surface will suffer from significant solar intrusion, which necessitates that the design of the FOV accounts for its impact. From Figure 5, which illustrates the Earth’s and the solar trajectory in the lunar sky, and Figure 6, it can be observed that during each orbital period, the Sun will transit once behind the Earth in the lunar sky. The minimum angle varies between 0.5° and 5° [49], indicating that solar intrusion is likely to occur in almost every orbital period. The occurrence of solar intrusion is related to the placement location of the Moon-based Earth observation multispectral camera on the lunar surface. On the western hemisphere of the Moon, solar intrusion will occur during the lunar forenoon, while on the eastern hemisphere, it will occur during the lunar afternoon. The duration of solar intrusion is not related to the latitude at which the sensor is placed.

**Figure 7:**
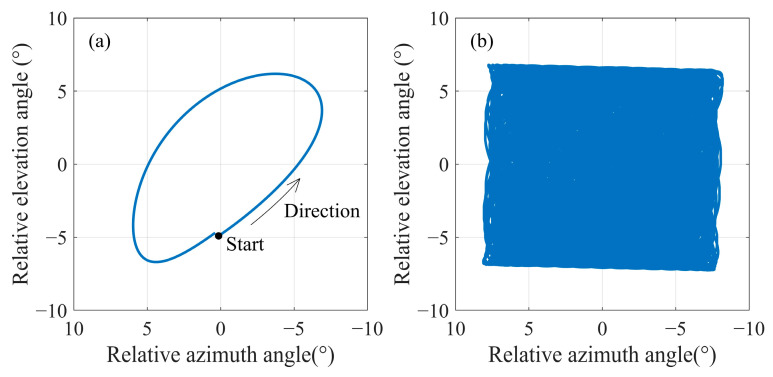
(**a**) The Earth’s trajectory during one orbital period. (**b**) The Earth’s trajectory from 2024 to 2044.

As shown in Figure 7, within an orbital period in the lunar sky, the Earth traces a trajectory resembling an elliptical path. The trajectory is not constant; influenced by lunar libration, each orbital period presents a distinct path, ultimately forming a rectangular envelope. This envelope reaches approximately 12° in the elevation angle of the sensor and about 14° in the azimuth angle. In other words, to design an FOV that encompasses the full range of the Earth’s motion during the 18.6-year cycle, an FOV not less than 14° would be required. Although such a design provides a larger observation range in the lunar sky, the FOV would be seven times the Earth’s apparent diameter. Assuming a 2k × 2k array detector at the focal plane, the spatial resolution at the nadir point would be 100 km, which would be insufficient for the observations of mesoscale atmospheric and oceanic phenomena on Earth. In addition, a larger FOV also increases the risk of solar intrusion, reducing the time available for Earth observation. Therefore, a larger FOV is not always preferable for Moon-based Earth observation sensors.

### 4.3. Impact of Turntable Performance on the Determination of the FOV

The Earth moves in the lunar sky over time. On the lunar surface, the sensors need to be mounted on a turntable to facilitate pointing towards Earth. The performance of the turntable, which affects the determination of the FOV, includes two aspects: one is the pointing accuracy, and the other is the setting of the adjustment temporal interval.

**Figure 8:**
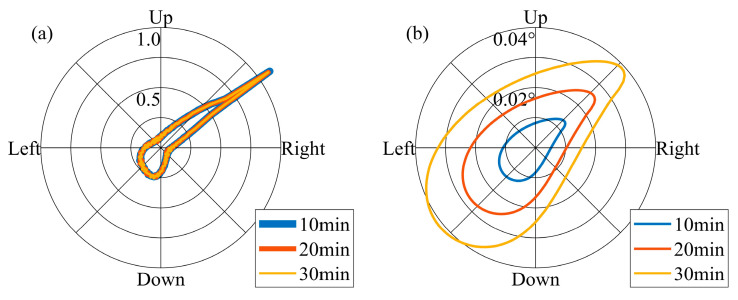
(**a**) PDF of the Earth’s movement direction and (**b**) the magnitude of its movement during one orbital period.

**Figure 9:**
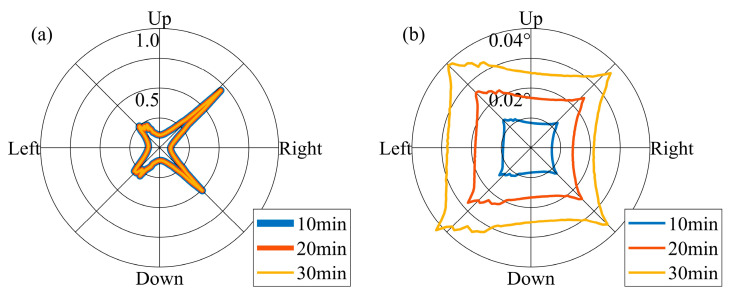
(**a**) PDF of the Earth’s movement direction and (**b**) the magnitude of its movement during an 18.6-year cycle.

Setting an appropriate temporal interval for adjusting the pointing vector is crucial. The design of the FOV must ensure that it encompasses the Earth’s movement throughout this specified duration. To analyze this point, it is necessary to clarify the direction of the Earth’s movement in the lunar sky and the magnitude of the Earth’s movement at different temporal intervals. As illustrated in Figure 7a, the direction of the Earth’s movement during one orbital period is not constant. Referring to the temporal sampling interval of the Moon-based Earth observation multispectral camera, we set 10 min, 20 min, and 30 min as the intervals for adjusting the pointing vector. We utilized the probability density function (PDF) to characterize the direction distribution of Earth’s movement during an orbital period. In order to prevent the value of PDFs from becoming excessively large or small, we treated PDFs as functions of radians and normalized them by setting the integral of the PDF from 0 to 2π equal to 1. In Figure 8a, it is evident that the direction of Earth’s movement within an orbital period does not change with respect to the pointing adjustment temporal intervals, and the direction is non-uniform. The reason for forming such a pattern is that, as shown in Figure 8a, when the Earth starts moving from the ‘start’ position, its trajectory is similar to a straight line, causing its directional samples to be concentrated within a particular direction. In terms of the magnitude of the Earth’s movement, it varies according to the distinct intervals. Over a 30 min interval, the Earth’s movement is approximately 0.04° (Figure 8b). Figure 9 further demonstrates the PDF of the Earth’s movement direction and the magnitude of its movement over an 18.6-year cycle. In terms of the direction of Earth’s movement, during the 18.6-year cycle, the Earth primarily moves towards four main directions. The reason can be attributed to the rectangle envelope pattern depicted in Figure 9b, which indicates a higher probability for the Earth to move in a straighter manner along four directions. In addition, in these four directions, the magnitude of Earth’s movement also reaches its peak. Consequently, at the 30 min interval level, the Earth’s movement varies by approximately an order of magnitude of 10^−2^°. Throughout the 18.6-year cycle, the Earth’s movement exhibits a lack of concentration in direction.

Pointing errors, which arise from a variety of sources including instrumental misalignment, the characteristics of rotation mechanisms, and the impact of lunar surface environmental impacts, can significantly affect the determination of the FOV of a Moon-based Earth observation multispectral camera. Since the sensor cannot point precisely towards the Earth and the Earth–Moon distance is very large, even minor pointing errors can cause significant movement of the Earth’s barycenter in the FOV. Consequently, it is necessary to design a slightly larger FOV to accommodate all extreme cases.

**Figure 10:**
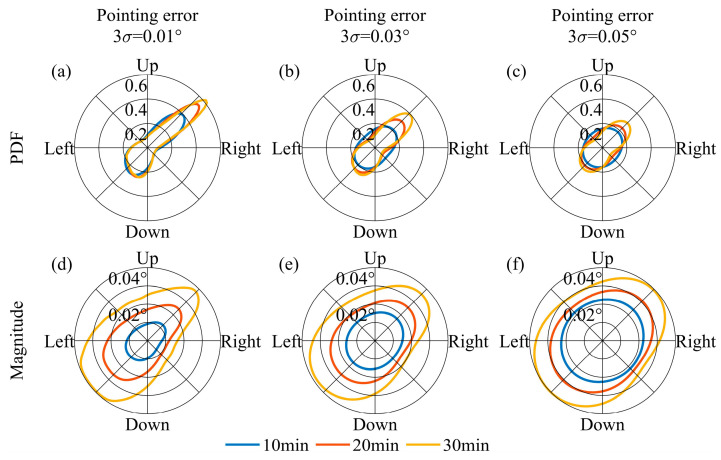
When the pointing adjustment temporal intervals were set to 10, 20, and 30 minutes, the PDF of the Earth’s movement direction and the magnitude of its movement across pointing errors during one orbital period. Subfigures (**a**–**c**) show the PDF curves as functions of radians, accounting for pointing errors interference, with the constraint that the integral of the PDFs from 0 to 2π is equal to 1. Subfigures (**d**–**f**) display the mathematical expectations of Earth’s offset magnitude along different directions while considering the interference of pointing errors. In subfigures (**a**,**d**), (**b**,**e**), (**c**,**f**), the upper limits of pointing errors are 0.01°, 0.03°, and 0.05° respectively.

**Figure 11:**
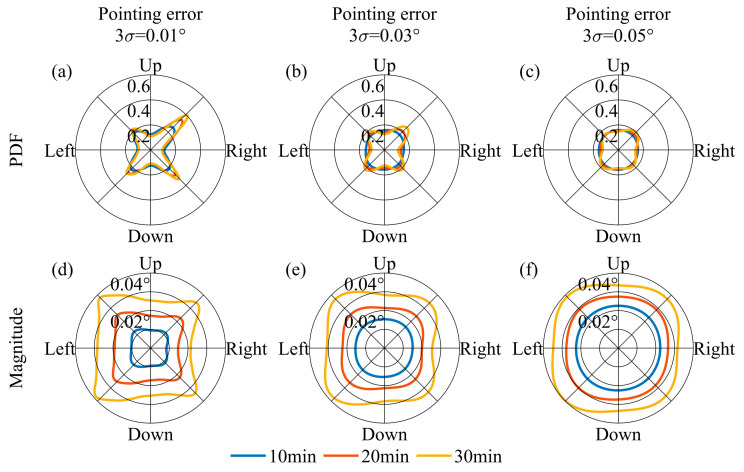
When the pointing adjustment temporal intervals were set to 10, 20, and 30 minutes, the PDF of the Earth’s movement direction and the magnitude of its movement across pointing errors during an 18.6-year cycle. The explanation for subfigures (**a**–**f**) is the same as in Figure 10.

We investigate the direction and magnitude of the movement of the Earth’s barycenter in the FOV when the pointing error is set 0.01°, 0.03°, and 0.05°, respectively. In this context, the pointing error is assumed to follow a normal distribution, with the specified values representing the 3*σ* condition. Figure 10 and Figure 11 show the movement of the Earth’s barycenter in the FOV caused by pointing errors. Due to the random nature of pointing errors’ directions, pointing errors significantly diminish the directionality of the Earth’s movement caused by pointing adjustment intervals. The greater the pointing error, the more pronounced the weakening effect on the directionality of Earth’s movement. As the pointing error increases, the directionality of Earth’s movement gradually diminishes when the effects of pointing errors approach or exceed the Earth’s movement within the pointing adjustment temporal intervals. In terms of the magnitude, the impact of different pointing errors on Earth’s movement is relatively similar. This is because the expected value of the pointing error is zero; the magnitude will not increase if the Earth’s movement caused by the pointing error is less than that during the pointing adjustment temporal interval.

**Figure 12:**
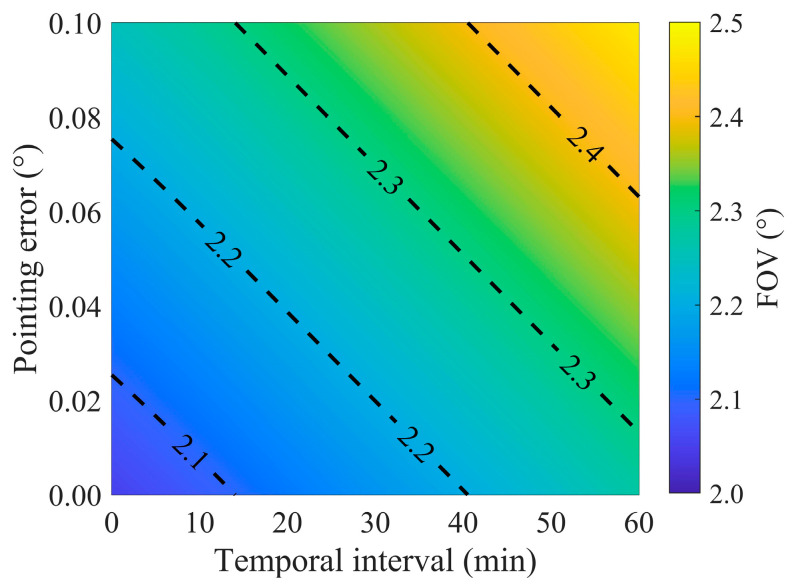
The maximum FOV needed to detect the Earth’s whole disk as a function of the pointing error and adjustment temporal interval.

Furthermore, we demonstrate the extreme FOV requirements under different pointing errors and pointing adjustment temporal intervals. It can be observed from Figure 12 that if designed with a pointing error of 0.05°, the pointing adjustment temporal interval should be less than 15 min, at which point the FOV can be set to 2.2°. However, as a short pointing adjustment temporal interval can significantly impact the lifespan of the turntable. It is suggested that, under the continuous observation requirements of a Moon-based Earth observation multispectral camera, to design a smaller FOV, efforts should be made to minimize pointing errors and extend the adjustment temporal intervals as much as possible.

## 5. Discussion

Currently, the design of the FOV in the fields of LEO and GEO satellites has reached a relatively mature stage. However, deploying a multispectral camera on the lunar surface presents unique challenges in terms of FOV design. The Earth–Moon distance is approximately 380,000 km, significantly exceeding the orbit altitude of LEO and GEO satellite. Furthermore, the Moon is a natural celestial body, and placing equipment at different locations on its surface yields varying observational performance [50,51].

This study reveals the characteristics of the Earth’s and the solar trajectory in the lunar sky from different locations on the lunar surface. The orientation of the Earth from different locations on the Moon, the Earth phase angle, and the envelope of the Earth’s trajectory in the lunar sky over the 18.6-year cycle are presented. Within the regions where the Earth is always visible, the Earth’s trajectory over an 18.6-year period forms an envelope of approximately 12° by 14°. Otherwise, the Earth will be obscured at certain time, and the sensor, having a lower elevation angle, will be more susceptible to stray light from the lunar surface. It is found that the Earth appears in a particular direction in the lunar sky when viewed from different positions on the lunar surface, which is determined by the longitude of the sensor’s position. Therefore, the FOV design must consider the trajectory patterns and the geometric relationships between the Earth, Moon, and Sun. This ensures the ability of the sensor to capture the necessary data effectively, minimizing the effects of lunar surface reflections or obstructions. For reference, the Apollo 16 Far-Ultraviolet Camera has an FOV of 20° [29], and the Chang’e-3 Ultraviolet Camera is designed with an FOV of 14.7° [28]. It is inferred that such designs not only cover the entire outer atmosphere of the Earth but also aim to cover the Earth’s entire trajectory within the observation period, thereby reducing the effort required for pointing adjustments of the turntable. Moreover, a thorough understanding of these celestial mechanics is crucial for planning observation schedules and optimizing the sensor’s operational parameters to align with the Earth’s visibility period.

This study analyzed the factors that must be considered in the design of the FOV at an Earth observation platform such as this. The primary objective of a Moon-based multispectral camera is to achieve observation of the Earth’s full disk. There are numerous constraints in energy and optics during the engineering implementation. Striving to achieve the smallest possible FOV is a goal pursued by engineers. Previous studies have not fully considered the extreme conditions [23,52]. This study, after considering the extreme case of Earth’s apparent diameter, further investigates the relationship between the Earth’s phase angle and its apparent diameter. When the Earth’s apparent diameter is large, the Earth’s full disk is also visible. From the perspective of observations during lunar day and lunar night, the Earth’s apparent diameter is only related to the Earth–Moon distance, with a period of a sidereal month, which is not synchronized with the lunar day–night cycle. Therefore, within the 18.6-year cycle, the moment when the Earth’s apparent diameter reaches its maximum can occur at any time during a synodic month, meaning that, even when choosing to observe only during the lunar day, it is not possible to reduce the Earth’s maximum apparent diameter. Consequently, this indicates that the FOV design must exceed the Earth’s maximum apparent diameter, which is 2.05°.

Another critical factor to ensure observation of the Earth’s full disk is the rotation mechanism of the turntable. The design of the FOV must account for the potential impacts of the rotation mechanism. The impact of the rotation mechanism on the FOV design is primarily manifested in two aspects: one is the temporal interval for its pointing adjustments, and the other is the pointing error. The sensor, when pointed in a stationary direction at a certain temporal interval, indicates that over the course of an 18.6-year cycle, the Earth’s movement within the FOV lacks a distinct directional pattern. With a 30 min interval, the magnitude of the Earth’s movement is approximately 0.04°. When considering the sensor’s pointing error, the Earth’s expected movement within the FOV depends on the relative magnitudes of the displacement caused by the sensor’s pointing accuracy and the movement over a given temporal interval. When the impact of pointing error exceeds that of the Earth’s movement within a given time interval, the influence of pointing error on the Earth’s expected movement increases relative to the Earth’s movement during that interval. Therefore, it is indicated that the rotation mechanism’s characteristics, such as pointing errors and the adjustment temporal interval, significantly influence the FOV design. Pointing accuracy and the adjustment temporal interval must be jointly designed to determine the FOV. The findings support the 2.5° FOV design adopted by Boyd et al. [19], suggesting that their design is well founded.

Moreover, the design of the FOV cannot be expanded indefinitely. Unlike the sensors onboard the platform at the Sun–Earth L1 point, which, due to its unique observe geometry, does not have to consider the issue of solar intrusion [49], the impact of solar intrusion must be taken into account in the Moon-based sensor design. The duration of solar intrusion is related to the sensor’s FOV, while the timing of solar intrusion is dependent on the location where the sensor is placed. Sensors equipped in the western hemisphere of the Moon experience solar intrusion during the lunar afternoon, whereas those in the eastern hemisphere experience it during the lunar forenoon. In contrast to previous studies that focus on ideal geometric analyses, it is worth noting that the effects of solar stray light must also consider whether the solar rays will strike the primary mirror, as this can also cause rapid temperature increases on the mirror. Therefore, the cutoff angle between the Sun and the sensor’s pointing direction should be significantly greater than half of the FOV angle, and it should also take into account the exit aperture size and the distance between the exit aperture and the primary mirror. Further work will focus on optimizing the optical system design in conjunction with the sensor’s FOV requirements to achieve better performance. Our study provides invaluable references for further optical design.

## 6. Conclusions

In this study, we conducted an analysis of the FOV for a Moon-based Earth observation multispectral camera. Our findings indicate that the FOV design must consider not only the Earth’s apparent diameter but also the impact of the pointing adjustment temporal intervals and the errors of the turntable should also be taken into account. We accounted for atmospheric refraction to calculate the Earth’s apparent diameter and found that, even at its maximum, a full phase of the Earth is visible, implying that the FOV should exceed this maximum value.

We analyzed the Earth’s and the solar trajectories in the lunar sky, noting that the Earth’s trajectory forms a pattern of several tens of degrees. This suggests that a 20° FOV could potentially cover the Earth’s trajectory; however, the FOV should still not be designed to be excessively large due to the impact of solar intrusion in every orbital period.

Moreover, the performance of the turntable is a critical factor in FOV design, with both pointing accuracy and adjustment intervals influencing the FOV. To achieve a smaller FOV for a Moon-based Earth observation multispectral camera, it is essential to minimize pointing errors and maximize the duration between adjustment temporal intervals whenever possible.

Given the importance of FOV design for future lunar missions, which significantly impacts optical and thermal control, this study provides a focused analysis of the unique challenges posed by a Moon-based platform. All these results contribute to a reasonable design of the sensor in future missions.

## Data Availability

The data presented in this study are available on request from the corresponding author.
